# *N*-{4-[4-(4-Fluoro­phen­yl)-1-methyl-2-[(*R*)-methyl­sulfin­yl]-1*H*-imidazol-5-yl]-2-pyridyl}acetamide dihydrate

**DOI:** 10.1107/S1600536809051873

**Published:** 2009-12-12

**Authors:** Stefanie Bühler, Dieter Schollmeyer, Dominik Hauser, Wolfgang Albrecht, Stefan Laufer

**Affiliations:** a Eberhard-Karls-University Tübingen, Auf der Morgenstelle 8, 72076 Tübingen, Germany; b University Mainz, Institut of Organic Chemistry, Duesbergweg 10-14, 55099 Mainz, Germany; c c-a-i-r biosciences GmbH, Paul-Ehrlich-Str. 15, 72076 Tübingen, Germany

## Abstract

In the crystal structure of the title compound, C_18_H_17_FN_4_O_2_S·2H_2_O, the organic mol­ecules are linked by inter­molecular O—H⋯O, O—H⋯N and N—H⋯O hydrogen bonds with the water mol­ecules, generating a three-dimensional network. The imidazole ring system forms a dihedral angle of 24.9 (2)° with the 4-fluoro­phenyl ring. The pyridine ring is oriented approximately perpendicular [72.24 (8)°] to the imidazole ring system.

## Related literature

For general background and the biological activity of chiral sulfoxides and their potential use as therapeutic agents, see: Jia *et al.*, (2004 ▶). Sulfoxide enanti­omers can differ in their pharmacodynamic and/or pharmacokinetic properties, see: Lu (2007 ▶). For the preparation of tri- and tetra­substituted 2-thio­imidazoles, see: Wagner *et al.* (2003 ▶); Laufer, Hauser, Domeyer *et al.* (2008 ▶); Laufer, Hauser & Liedtke (2008 ▶).
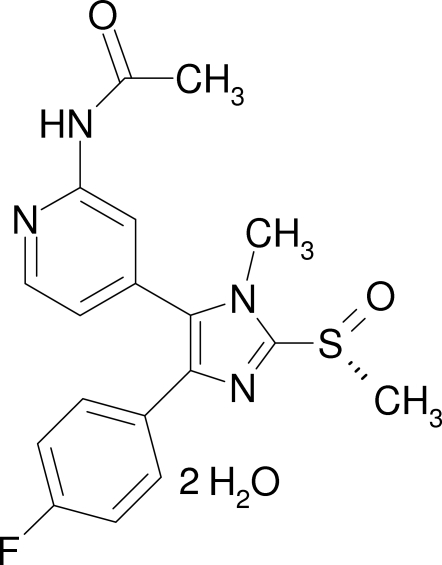


## Experimental

### 

#### Crystal data

C_18_H_17_FN_4_O_2_S·2H_2_O
*M* *_r_* = 408.45
Orthorhombic, 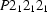

*a* = 5.8178 (2) Å
*b* = 14.3233 (5) Å
*c* = 23.3694 (9) Å
*V* = 1947.37 (12) Å^3^
*Z* = 4
Mo *K*α radiation
μ = 0.21 mm^−1^
*T* = 193 K
0.50 × 0.10 × 0.10 mm

#### Data collection

Bruker SMART APEXII diffractometer
10703 measured reflections
4628 independent reflections
2634 reflections with *I* > 2σ(*I*)
*R*_int_ = 0.089

#### Refinement

*R*[*F* ^2^ > 2σ(*F* ^2^)] = 0.046
*wR*(*F* ^2^) = 0.099
*S* = 0.84
4628 reflections
256 parameters
H-atom parameters constrained
Δρ_max_ = 0.27 e Å^−3^
Δρ_min_ = −0.30 e Å^−3^
Absolute structure: Flack (1983 ▶), 1949 Friedel pairs
Flack parameter: −0.08 (11)

### 

Data collection: *APEX2* (Bruker, 2006 ▶); cell refinement: *SAINT* (Bruker, 2006 ▶); data reduction: *SAINT*; program(s) used to solve structure: *SIR97* (Altomare *et al.*, 1999 ▶); program(s) used to refine structure: *SHELXL97* (Sheldrick, 2008 ▶); molecular graphics: *PLATON* (Spek, 2009 ▶); software used to prepare material for publication: *PLATON*.

## Supplementary Material

Crystal structure: contains datablocks I, global. DOI: 10.1107/S1600536809051873/nc2170sup1.cif

Structure factors: contains datablocks I. DOI: 10.1107/S1600536809051873/nc2170Isup2.hkl

Additional supplementary materials:  crystallographic information; 3D view; checkCIF report

## Figures and Tables

**Table 1:** Hydrogen-bond geometry (Å, °)

*D*—H⋯*A*	*D*—H	H⋯*A*	*D*⋯*A*	*D*—H⋯*A*
N24—H24⋯O15^i^	0.91	2.08	2.983 (3)	177
O1*L*—H1*L*⋯O26^ii^	0.94	1.90	2.825 (3)	169
O1*L*—H2*L*⋯O15	0.85	2.00	2.775 (3)	151
O2*L*—H3*L*⋯O1*L*	0.84	1.90	2.743 (4)	177
O2*L*—H4*L*⋯N2	0.84	2.51	3.193 (3)	138

Symmetry codes: (i) 
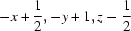
; (ii) 
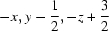
.

**Fig. 1.**
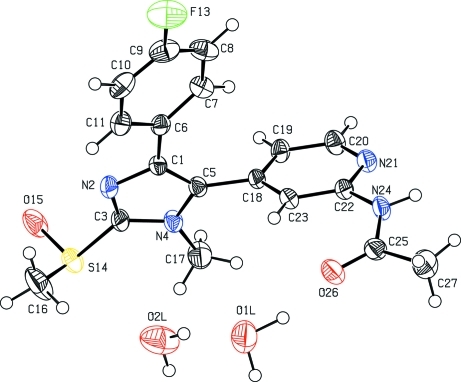
View of compound I. Displacement ellipsoids are drawn at the 50% probability level.

## supplementary crystallographic information

### Comment

Many chiral sulfoxides exhibit interesting biological activities and show promise as therapeutic agents (Jia *et al.*, 2004). The title compound, *N*-(4-(4-(4-fluorophenyl)-1-methyl-2-((*R*)-methylsulfinyl)-1*H*-imidazol-5-yl) pyridin-2-yl)acetamide, was prepared in the course of our studies on tri- and tetrasubstituted 2-thioimidazoles as p38 mitogen-activated protein (MAP) kinase inhibitors (Wagner *et al.*, 2003, Laufer, Hauser, Domeyer *et al.*,2008); Laufer, Hauser & Liedtke, 2008). The oxidation of the sulfide *N*-(4-(4-(4-fluorophenyl)-1-methyl-2-(methylthio)-1*H*-imidazol-5-yl)pyridin-2- yl)acetamide with sodium periodate as oxidant yields the corresponding sulfoxide *N*-(4-(4-(4-fluorophenyl)-1-methyl-2-(methylsulfinyl)-1*H*-imidazol-5-yl) pyridin-2-yl)acetamide. This compound has a centre of asymmetry in the sulfur atom meaning that two sulfoxide enantiomers exist, which can differ in pharmacodynamic and/or pharmacokinetic properties (Lu, 2007). The both enantiomers were separated by enantioselective preparative HPLC. To identify the absolute configurations of the enantiomers according to the Cahn-Ingold-Prelog-nomenclature X-ray analysis was used. In this article we present the X-ray data of the first eluted enantiomer I. The analysis of the crystal structure shows that the N24 amino- group of the acetamide moiety of the one imidazole-molecule interact with another imidazole ring system by the building of one intermolecular hydrogen bond N—H···O to the sulfoxide oxygen-atom O15, whereas the O15···H24 distance is 2.08 Å. Furthermore, the carbonyl-oxygen atom O26 of the acetamide-moiety of a third imidazole molecule is linked over two water molecules to the sulfoxide oxygen-atom O15 and to the nitrogen atom N2 of the imidazole core by the building of intermolecular O1L—H1L···O26 (1.90 Å), O1L—H2L···O15 (2.00 Å) and O2L—H4L···N2 (2.51 Å) hydrogen bonds. The imidazole ring system forms a dihedral angle of 24.9 (1)° to the 4-fluorophenyl ring. The pyridine ring is oriented approximately perpendicular (72.24 (8)°) to the imidazole ring system. According to the Cahn-Ingold-Prelog-nomenclature the first eluted enantiomer I is (*R*)-configurated.

### Experimental

*N*-(4-(4-(4-fluorophenyl)-1-methyl-2-(methylthio)-1*H*-imidazol-5-yl)pyridin-2- yl)acetamide (3.56 g, 10 mmol) was dissolved in a mixture of THF (30 ml) and acetone (58 ml), and water (45 ml) was added. The mixture was stirred at T = 313 K, and an aqueous solution of sodium periodate (4.01 g, 18.4 mmol in 65 ml water) was added dropwise. The reaction mixture was heated at T = 338 K. The progress was again monitored until HPLC analysis (C8 Betasil, MeOH/ 0.01 *M* KH_2_PO_4_, pH = 2,3) indicated complete conversion. After cooling to rt, the organic solvents were removed under reduced pressure and a precipitate dropped down, which was filtered off and washed with water and isopropylether. The crude product was purified by recrystallization from ethylacetate to yield 3.02 g (81.2%) of the colorless racemic product *N*-(4-(4-(4-fluorophenyl)-1-methyl-2-(methylsulfinyl)-1*H*-imidazol-5-yl)pyridin-2-yl)acetamide. The both enantiomers were isolated by enantioselective preparative HPLC (Daicel CHIRALPAK AD, CH_2_Cl_2_ / MeOH/ TEA 98: 2: 0.1). Suitable crystals of the first eluted enantiomer I for X-ray were obtained by slow evaporation at T = 298 K of a solution of ethanol.

### Refinement

Hydrogen atoms attached to carbons were placed at calculated positions with C—H = 0.95 Å (aromatic) or 0.98–0.99 Å (*sp*^3^ C-atom). Hydrogen atoms attached to nitrogen and oxygen were located in diff. Fourier maps. All H atoms were refined in the riding-model approximation with isotropic displacement parameters (set at 1.2–1.5 times of the *U*_eq_ of the parent atom). The absolute structure was determined on the basis of 1949 Friedel pairs.

### Crystal data

**Table:**

C_18_H_17_FN_4_O_2_S·2H_2_O	*F*(000) = 856
*M**_r_* = 408.45	*D*_x_ = 1.393 Mg m^−^^3^
Orthorhombic, *P*2_1_2_1_2_1_	Mo *K*α radiation, λ = 0.71069 Å
Hall symbol: P 2ac 2ab	Cell parameters from 1669 reflections
*a* = 5.8178 (2) Å	θ = 2.9–23.5°
*b* = 14.3233 (5) Å	µ = 0.21 mm^−^^1^
*c* = 23.3694 (9) Å	*T* = 193 K
*V* = 1947.37 (12) Å^3^	Needle, colourless
*Z* = 4	0.50 × 0.10 × 0.10 mm

### Data collection

**Table:**

Bruker SMART APEXII diffractometer	2634 reflections with *I* > 2σ(*I*)
Radiation source: sealed Tube	*R*_int_ = 0.089
graphite	θ_max_ = 27.9°, θ_min_ = 1.7°
CCD scan	*h* = −7→7
10703 measured reflections	*k* = −18→15
4628 independent reflections	*l* = −24→30

### Refinement

**Table:**

Refinement on *F*^2^	Secondary atom site location: difference Fourier map
Least-squares matrix: full	Hydrogen site location: inferred from neighbouring sites
*R*[*F*^2^ > 2σ(*F*^2^)] = 0.046	H-atom parameters constrained
*wR*(*F*^2^) = 0.099	*w* = 1/[σ^2^(*F*_o_^2^) + (0.0293*P*)^2^] where *P* = (*F*_o_^2^ + 2*F*_c_^2^)/3
*S* = 0.84	(Δ/σ)_max_ = 0.001
4628 reflections	Δρ_max_ = 0.27 e Å^−^^3^
256 parameters	Δρ_min_ = −0.29 e Å^−^^3^
0 restraints	Absolute structure: Flack (1983), 1949 Friedel pairs
Primary atom site location: structure-invariant direct methods	Flack parameter: −0.08 (11)

### Special details

**Table:**

Geometry. All e.s.d.'s (except the e.s.d. in the dihedral angle between two l.s. planes) are estimated using the full covariance matrix. The cell e.s.d.'s are taken into account individually in the estimation of e.s.d.'s in distances, angles and torsion angles; correlations between e.s.d.'s in cell parameters are only used when they are defined by crystal symmetry. An approximate (isotropic) treatment of cell e.s.d.'s is used for estimating e.s.d.'s involving l.s. planes.
Refinement. Refinement of *F*^2^ against ALL reflections. The weighted *R*-factor *wR* and goodness of fit *S* are based on *F*^2^, conventional *R*-factors *R* are based on *F*, with *F* set to zero for negative *F*^2^. The threshold expression of *F*^2^ > σ(*F*^2^) is used only for calculating *R*-factors(gt) *etc*. and is not relevant to the choice of reflections for refinement. *R*-factors based on *F*^2^ are statistically about twice as large as those based on *F*, and *R*- factors based on ALL data will be even larger.

### Fractional atomic coordinates and isotropic or equivalent isotropic displacement parameters (Å^2^)

**Table:**

	*x*	*y*	*z*	*U*_iso_*/*U*_eq_	
C1	−0.0028 (5)	0.38294 (18)	0.67677 (10)	0.0247 (6)	
N2	0.0086 (5)	0.38684 (16)	0.73573 (9)	0.0301 (6)	
C3	0.1670 (5)	0.44957 (18)	0.74681 (10)	0.0299 (7)	
N4	0.2625 (4)	0.48701 (15)	0.69850 (7)	0.0276 (5)	
C5	0.1496 (5)	0.44518 (18)	0.65318 (10)	0.0244 (6)	
C6	−0.1622 (5)	0.31718 (18)	0.64897 (11)	0.0265 (7)	
C7	−0.1212 (6)	0.2827 (2)	0.59361 (11)	0.0334 (7)	
H7	0.0139	0.3008	0.5736	0.040*	
C8	−0.2761 (6)	0.2227 (2)	0.56802 (13)	0.0406 (8)	
H8	−0.2504	0.2003	0.5303	0.049*	
C9	−0.4667 (6)	0.1961 (2)	0.59797 (14)	0.0428 (9)	
C10	−0.5117 (6)	0.2250 (2)	0.65250 (13)	0.0391 (8)	
H10	−0.6438	0.2036	0.6725	0.047*	
C11	−0.3591 (5)	0.2864 (2)	0.67776 (12)	0.0326 (7)	
H11	−0.3885	0.3082	0.7154	0.039*	
F13	−0.6195 (4)	0.13680 (13)	0.57211 (9)	0.0687 (6)	
S14	0.25518 (17)	0.48524 (5)	0.81652 (3)	0.0404 (2)	
O15	0.3625 (4)	0.40219 (16)	0.84497 (7)	0.0490 (6)	
C16	−0.0249 (7)	0.4982 (3)	0.84489 (11)	0.0636 (12)	
H16C	−0.0151	0.5104	0.8861	0.095*	
H16B	−0.1127	0.4408	0.8383	0.095*	
H16A	−0.1020	0.5506	0.8260	0.095*	
C17	0.4374 (6)	0.5604 (2)	0.69480 (12)	0.0439 (9)	
H17A	0.4180	0.6041	0.7267	0.066*	
H17B	0.4205	0.5940	0.6585	0.066*	
H17C	0.5906	0.5321	0.6967	0.066*	
C18	0.2057 (5)	0.47072 (17)	0.59317 (9)	0.0244 (6)	
C19	0.4114 (5)	0.44302 (19)	0.56902 (11)	0.0285 (7)	
H19	0.5209	0.4092	0.5909	0.034*	
C20	0.4555 (6)	0.4653 (2)	0.51252 (11)	0.0331 (7)	
H20	0.5962	0.4448	0.4961	0.040*	
N21	0.3106 (4)	0.51404 (17)	0.47977 (8)	0.0294 (6)	
C22	0.1152 (5)	0.54307 (19)	0.50371 (11)	0.0262 (7)	
C23	0.0530 (5)	0.52212 (19)	0.56027 (10)	0.0264 (6)	
H23	−0.0896	0.5426	0.5756	0.032*	
N24	−0.0213 (4)	0.59582 (16)	0.46634 (9)	0.0314 (6)	
H24	0.0221	0.5970	0.4291	0.047*	
C25	−0.1937 (6)	0.65639 (19)	0.47982 (12)	0.0327 (7)	
O26	−0.2713 (4)	0.66543 (13)	0.52821 (8)	0.0405 (5)	
C27	−0.2867 (7)	0.7123 (2)	0.43046 (12)	0.0530 (10)	
H27A	−0.2501	0.7784	0.4361	0.080*	
H27C	−0.4538	0.7044	0.4284	0.080*	
H27B	−0.2166	0.6904	0.3948	0.080*	
O1L	0.2647 (6)	0.21319 (16)	0.85452 (9)	0.0726 (8)	
H1L	0.2717	0.2057	0.8945	0.109*	
H2L	0.2448	0.2723	0.8557	0.109*	
O2L	0.0400 (5)	0.16661 (18)	0.75536 (11)	0.0795 (9)	
H3L	0.1051	0.1806	0.7863	0.119*	
H4L	−0.0357	0.2127	0.7435	0.119*	

### Atomic displacement parameters (Å^2^)

**Table:**

	*U*^11^	*U*^22^	*U*^33^	*U*^12^	*U*^13^	*U*^23^
C1	0.0296 (16)	0.0238 (15)	0.0207 (13)	0.0027 (13)	−0.0021 (14)	0.0000 (11)
N2	0.0400 (16)	0.0291 (14)	0.0211 (11)	−0.0024 (12)	−0.0021 (12)	0.0038 (10)
C3	0.0408 (19)	0.0302 (15)	0.0186 (13)	−0.0020 (14)	−0.0058 (14)	0.0009 (11)
N4	0.0324 (13)	0.0299 (12)	0.0206 (10)	−0.0038 (13)	−0.0037 (12)	0.0004 (9)
C5	0.0266 (17)	0.0261 (14)	0.0206 (13)	0.0042 (13)	−0.0028 (13)	0.0002 (11)
C6	0.0312 (19)	0.0238 (14)	0.0245 (13)	0.0029 (13)	−0.0061 (14)	0.0038 (11)
C7	0.0332 (18)	0.0328 (17)	0.0341 (16)	0.0031 (16)	−0.0017 (15)	−0.0034 (13)
C8	0.044 (2)	0.0349 (17)	0.0434 (16)	0.0021 (19)	−0.0093 (19)	−0.0141 (14)
C9	0.036 (2)	0.0325 (19)	0.060 (2)	−0.0051 (17)	−0.0175 (19)	−0.0050 (16)
C10	0.0320 (19)	0.0342 (18)	0.0512 (19)	−0.0045 (16)	−0.0054 (17)	0.0131 (15)
C11	0.0333 (18)	0.0325 (17)	0.0318 (15)	0.0013 (15)	−0.0020 (15)	0.0071 (13)
F13	0.0562 (14)	0.0548 (13)	0.0950 (15)	−0.0195 (12)	−0.0165 (14)	−0.0227 (12)
S14	0.0586 (5)	0.0411 (4)	0.0215 (3)	−0.0017 (5)	−0.0099 (4)	−0.0032 (3)
O15	0.0636 (17)	0.0592 (15)	0.0243 (10)	0.0236 (14)	−0.0124 (11)	−0.0030 (10)
C16	0.072 (3)	0.091 (3)	0.0272 (15)	0.030 (3)	−0.0050 (18)	−0.0117 (18)
C17	0.048 (2)	0.046 (2)	0.0385 (17)	−0.0163 (17)	−0.0022 (17)	0.0006 (14)
C18	0.0316 (18)	0.0237 (14)	0.0180 (12)	−0.0032 (14)	−0.0011 (12)	−0.0002 (10)
C19	0.0288 (17)	0.0322 (17)	0.0243 (14)	0.0034 (14)	−0.0031 (14)	0.0050 (12)
C20	0.0310 (18)	0.040 (2)	0.0288 (15)	0.0034 (15)	0.0038 (14)	0.0011 (13)
N21	0.0311 (15)	0.0318 (13)	0.0254 (11)	0.0066 (12)	0.0012 (11)	0.0013 (10)
C22	0.0313 (18)	0.0258 (16)	0.0214 (13)	−0.0029 (14)	−0.0005 (13)	−0.0007 (11)
C23	0.0289 (17)	0.0288 (16)	0.0214 (13)	0.0013 (14)	0.0018 (12)	−0.0020 (12)
N24	0.0385 (16)	0.0373 (15)	0.0185 (11)	0.0101 (14)	0.0004 (12)	0.0032 (10)
C25	0.037 (2)	0.0295 (16)	0.0318 (15)	0.0049 (15)	−0.0035 (15)	−0.0010 (12)
O26	0.0463 (14)	0.0462 (12)	0.0289 (10)	0.0108 (13)	0.0015 (12)	−0.0037 (9)
C27	0.065 (3)	0.052 (2)	0.0415 (17)	0.026 (2)	−0.006 (2)	0.0082 (15)
O1L	0.105 (2)	0.0615 (16)	0.0516 (13)	0.0245 (19)	0.0045 (17)	−0.0070 (12)
O2L	0.086 (2)	0.0676 (18)	0.0850 (18)	−0.0002 (17)	−0.0200 (19)	0.0271 (15)

### Geometric parameters (Å, °)

**Table:**

C1—C5	1.373 (4)	C17—H17A	0.9800
C1—N2	1.380 (3)	C17—H17B	0.9800
C1—C6	1.473 (4)	C17—H17C	0.9800
N2—C3	1.313 (4)	C18—C19	1.381 (4)
C3—N4	1.368 (3)	C18—C23	1.386 (4)
C3—S14	1.783 (3)	C19—C20	1.382 (3)
N4—C5	1.383 (3)	C19—H19	0.9500
N4—C17	1.465 (4)	C20—N21	1.336 (3)
C5—C18	1.485 (3)	C20—H20	0.9500
C6—C11	1.400 (4)	N21—C22	1.333 (3)
C6—C7	1.405 (4)	C22—N24	1.402 (3)
C7—C8	1.382 (4)	C22—C23	1.403 (3)
C7—H7	0.9500	C23—H23	0.9500
C8—C9	1.365 (5)	N24—C25	1.363 (4)
C8—H8	0.9500	N24—H24	0.9072
C9—C10	1.365 (4)	C25—O26	1.224 (3)
C9—F13	1.370 (4)	C25—C27	1.505 (4)
C10—C11	1.382 (4)	C27—H27A	0.9800
C10—H10	0.9500	C27—H27C	0.9800
C11—H11	0.9500	C27—H27B	0.9800
S14—O15	1.499 (2)	O1L—H1L	0.9410
S14—C16	1.769 (4)	O1L—H2L	0.8545
C16—H16C	0.9800	O2L—H3L	0.8400
C16—H16B	0.9800	O2L—H4L	0.8400
C16—H16A	0.9800		
			
C5—C1—N2	110.1 (2)	H16C—C16—H16A	109.5
C5—C1—C6	130.1 (2)	H16B—C16—H16A	109.5
N2—C1—C6	119.8 (2)	N4—C17—H17A	109.5
C3—N2—C1	105.0 (2)	N4—C17—H17B	109.5
N2—C3—N4	113.0 (2)	H17A—C17—H17B	109.5
N2—C3—S14	125.3 (2)	N4—C17—H17C	109.5
N4—C3—S14	121.7 (2)	H17A—C17—H17C	109.5
C3—N4—C5	105.6 (2)	H17B—C17—H17C	109.5
C3—N4—C17	127.7 (2)	C19—C18—C23	118.7 (2)
C5—N4—C17	126.5 (2)	C19—C18—C5	120.3 (2)
C1—C5—N4	106.3 (2)	C23—C18—C5	120.9 (2)
C1—C5—C18	132.9 (2)	C18—C19—C20	119.0 (3)
N4—C5—C18	120.8 (2)	C18—C19—H19	120.5
C11—C6—C7	118.1 (3)	C20—C19—H19	120.5
C11—C6—C1	120.3 (2)	N21—C20—C19	123.4 (3)
C7—C6—C1	121.6 (3)	N21—C20—H20	118.3
C8—C7—C6	120.4 (3)	C19—C20—H20	118.3
C8—C7—H7	119.8	C22—N21—C20	117.4 (2)
C6—C7—H7	119.8	N21—C22—N24	112.9 (2)
C9—C8—C7	118.8 (3)	N21—C22—C23	123.3 (3)
C9—C8—H8	120.6	N24—C22—C23	123.8 (3)
C7—C8—H8	120.6	C18—C23—C22	118.1 (3)
C10—C9—C8	123.4 (3)	C18—C23—H23	121.0
C10—C9—F13	118.4 (3)	C22—C23—H23	121.0
C8—C9—F13	118.3 (3)	C25—N24—C22	128.0 (2)
C9—C10—C11	117.9 (3)	C25—N24—H24	114.5
C9—C10—H10	121.0	C22—N24—H24	116.8
C11—C10—H10	121.0	O26—C25—N24	123.5 (2)
C10—C11—C6	121.4 (3)	O26—C25—C27	121.3 (3)
C10—C11—H11	119.3	N24—C25—C27	115.2 (3)
C6—C11—H11	119.3	C25—C27—H27A	109.5
O15—S14—C16	107.50 (16)	C25—C27—H27C	109.5
O15—S14—C3	107.32 (12)	H27A—C27—H27C	109.5
C16—S14—C3	96.18 (15)	C25—C27—H27B	109.5
S14—C16—H16C	109.5	H27A—C27—H27B	109.5
S14—C16—H16B	109.5	H27C—C27—H27B	109.5
H16C—C16—H16B	109.5	H1L—O1L—H2L	94.9
S14—C16—H16A	109.5	H3L—O2L—H4L	109.5
			
C5—C1—N2—C3	−0.7 (3)	F13—C9—C10—C11	−178.6 (3)
C6—C1—N2—C3	178.7 (3)	C9—C10—C11—C6	−1.1 (4)
C1—N2—C3—N4	−0.4 (3)	C7—C6—C11—C10	−0.9 (4)
C1—N2—C3—S14	179.4 (2)	C1—C6—C11—C10	179.8 (3)
N2—C3—N4—C5	1.4 (3)	N2—C3—S14—O15	64.5 (3)
S14—C3—N4—C5	−178.5 (2)	N4—C3—S14—O15	−115.7 (2)
N2—C3—N4—C17	177.9 (3)	N2—C3—S14—C16	−46.0 (3)
S14—C3—N4—C17	−2.0 (4)	N4—C3—S14—C16	133.8 (3)
N2—C1—C5—N4	1.5 (3)	C1—C5—C18—C19	−107.8 (4)
C6—C1—C5—N4	−177.8 (3)	N4—C5—C18—C19	71.7 (3)
N2—C1—C5—C18	−178.9 (3)	C1—C5—C18—C23	71.8 (4)
C6—C1—C5—C18	1.8 (5)	N4—C5—C18—C23	−108.7 (3)
C3—N4—C5—C1	−1.7 (3)	C23—C18—C19—C20	−1.7 (4)
C17—N4—C5—C1	−178.2 (3)	C5—C18—C19—C20	177.9 (3)
C3—N4—C5—C18	178.7 (2)	C18—C19—C20—N21	1.2 (4)
C17—N4—C5—C18	2.1 (4)	C19—C20—N21—C22	0.8 (4)
C5—C1—C6—C11	−155.5 (3)	C20—N21—C22—N24	178.2 (2)
N2—C1—C6—C11	25.2 (4)	C20—N21—C22—C23	−2.3 (4)
C5—C1—C6—C7	25.2 (5)	C19—C18—C23—C22	0.3 (4)
N2—C1—C6—C7	−154.0 (3)	C5—C18—C23—C22	−179.3 (2)
C11—C6—C7—C8	2.1 (4)	N21—C22—C23—C18	1.8 (4)
C1—C6—C7—C8	−178.6 (3)	N24—C22—C23—C18	−178.8 (3)
C6—C7—C8—C9	−1.3 (4)	N21—C22—N24—C25	−160.2 (3)
C7—C8—C9—C10	−0.8 (5)	C23—C22—N24—C25	20.3 (4)
C7—C8—C9—F13	179.8 (3)	C22—N24—C25—O26	−8.5 (5)
C8—C9—C10—C11	2.0 (5)	C22—N24—C25—C27	172.0 (3)

### Hydrogen-bond geometry (Å, °)

**Table:**

*D*—H···*A*	*D*—H	H···*A*	*D*···*A*	*D*—H···*A*
N24—H24···O15^i^	0.91	2.08	2.983 (3)	177
O1L—H2L···O15	0.85	2.00	2.775 (3)	151
O1L—H1L···O26^ii^	0.94	1.90	2.825 (3)	169
O2L—H3L···O1L	0.84	1.90	2.743 (4)	177
O2L—H4L···N2	0.84	2.51	3.193 (3)	138

Symmetry codes: (i) −*x*+1/2, −*y*+1, *z*−1/2; (ii) −*x*, *y*−1/2, −*z*+3/2.
